# Structural Insight into Polymerase Mechanism via a Chiral Center Generated with a Single Selenium Atom

**DOI:** 10.3390/ijms242115758

**Published:** 2023-10-30

**Authors:** Tong Qin, Bei Hu, Qianwei Zhao, Yali Wang, Shaoxin Wang, Danyan Luo, Jiazhen Lyu, Yiqing Chen, Jianhua Gan, Zhen Huang

**Affiliations:** 1 Key Laboratory of Bio-Resource and Eco-Environment of Ministry of Education, College of Life Sciences, Sichuan University, Chengdu 610064, China; qintong_7@scu.edu.cn (T.Q.); hubei@scu.edu.cn (B.H.); qianwzhao@zzu.edu.cn (Q.Z.); wangshaoxin77@stu.scu.edu.cn (S.W.); lyujiazhen@nsmc.edu.cn (J.L.); 2 Henan Institute of Medical and Pharmaceutical Sciences, Zhengzhou University, Zhengzhou 450000, China; 3 College of Bioengineering, Sichuan University of Science and Engineering, Yibin 644000, China; ylwang@scu.edu.cn; 4 SeNA Research Institute and Szostak-CDHT Large Nucleic Acid Institute, Chengdu 618000, China; ldy@largenucleicacid.org; 5 Shanghai Public Health Clinical Center, State Key Laboratory of Genetic Engineering, Collaborative Innovation Center of Genetics and Development, Department of Biochemistry and Biophysics, School of Life Sciences, Fudan University, Shanghai 200438, China; yiqing.chen@rnacure.com; 6 State Key Laboratory of Southwestern Chinese Medicine Resources, Chengdu University of Traditional Chinese Medicine, Chengdu 610000, China

**Keywords:** polymerase, single selenium atom, phosphodiester bond formation, configuration inversion, metal ions

## Abstract

DNA synthesis catalyzed by DNA polymerase is essential for all life forms, and phosphodiester bond formation with phosphorus center inversion is a key step in this process. Herein, by using a single-selenium-atom-modified dNTP probe, we report a novel strategy to visualize the reaction stereochemistry and catalysis. We capture the before- and after-reaction states and provide explicit evidence of the center inversion and in-line attacking S_N_2 mechanism of DNA polymerization, while solving the diastereomer absolute configurations. Further, our kinetic and thermodynamic studies demonstrate that in the presence of Mg^2+^ ions (or Mn^2+^), the binding affinity (*K*_m_) and reaction selectivity (*k*_cat_/*K*_m_) of dGTPαSe-Rp were 51.1-fold (or 19.5-fold) stronger and 21.8-fold (or 11.3-fold) higher than those of dGTPαSe-Sp, respectively, indicating that the diastereomeric Se-Sp atom was quite disruptive of the binding and catalysis. Our findings reveal that the third metal ion is much more critical than the other two metal ions in both substrate recognition and bond formation, providing insights into how to better design the polymerase inhibitors and discover the therapeutics.

## 1. Introduction

As the major carrier of genetic information, DNA must be replicated, since it is essential for maintaining the genetic materials of life forms [1]. Genetic information is transferred from one generation to the next through DNA synthesis catalyzed by DNA polymerase [2,3,4], which has been an intensive research area for many decades. Since Steitz resolved the first polymerase structure in 1985, structures of more than 30 DNA polymerases have been elucidated, including 17 in humans and 13 in other species [5,6,7,8]. Almost all polymerases share a similar right-handed-like architecture and a catalytic mechanism for new phosphodiester bond formation [9,10].

The classical two-metal-ion mechanism suggested that with the assistance of two conserved aspartate residues and a carbonyl oxygen, two metal ions less than 4 Å apart mediate the phosphodiester bond formation [11,12,13,14]. In this chemical reaction, metal-ion A (MeA) is a catalytic ion and decreases the p*K*a of 3′-OH, facilitating its attack on the α-phosphate of the incoming dNTP, and metal-ion B (MeB) stabilizes the dNTP-DNA-polymerase complex and increases the electrophilicity of the phosphorus center [15,16,17]. Via the time-resolved X-ray crystallography [18,19,20,21,22], recently in Pol η, β, λ, and μ, a third metal ion (metal C, MeC) has been suggested to facilitate the bond formation [23], though its detailed catalysis role is still not well understood.

Although kinetics, time-resolved crystallography, and modified nucleotides have made considerable contributions to the mechanistic studies of polymerase [24,25,26,27,28,29], there are many controversies about the mechanisms of the metal-ion-mediated catalysis [30,31], especially the third metal ion. In addition, given their important roles in genetic information storage and transfer, in principle, polymerases are potential targets for efficient drug designs to treat many diseases [32,33,34,35,36]. To clearly understand the phosphodiester-bond-formation mechanism and the metal-ion catalysis of polymerases, we designed modified dNTP substrates with a chiral α-phosphorus center (α-P center) using a single selenium atom, without affecting the overall and local structures, to explore the absolute configuration inversion and probe the mechanisms of the metal ion–oxygen interactions. Selenium (atomic radius: 1.16 Å) in the same elemental family as oxygen (atomic radius: 0.73 Å) is an ideal heavy atom for structure determination and electron-density mapping [37,38,39,40], which can atom-specifically disrupt and probe the interactions between the O atoms and metal ions. Therefore, in principle, it should be much easier to observe the inversion mechanism and the oxygen-participated catalysis using the selenium strategy than the time-resolved method. To provide a molecular basis for designing better drugs targeting polymerases, African swine fever virus DNA Polymerase X (Pol X) was used as a model for the study. As the simplest DNA polymerase identified to date, Pol X belongs to the polymerase X family and is constituted of 174 amino acids [7,41,42]. Lacking two DNA-binding domains [43,44] and a proofread 3′-5′-exonuclease activity may make Pol X tolerate the dNTP modifications.

By taking advantage of this polymerase, herein, we report our study of the stereochemistry and reaction mechanism of DNA synthesis using a simple strategy with Se-atom-modified dGTPs. Firstly, dGTPαSe diastereomers were synthesized and separated by reversed-phase-HPLC as peak 1 (dGTPαSe-I, eluted first on HPLC) and peak 2 (dGTPαSe-II, eluted later) [45], and their absolute configurations on the α-P center were determined as Rp and Sp, respectively, by X-ray crystallography. Further, the configurations of polymerization products (using dGTPαSe-I and dGTPαSe-II) were determined as Sp and Rp, respectively. By comparing the absolute configurations (Rp and Sp) at the P-center in the before- and after-reaction states (easily visualized by the single Se atom), we observed the inversion at the α-P center in the polymerization reaction, thereby establishing a new strategy to easily illustrate the mechanism of the polymerization reaction. Furthermore, in one single crystal with Se modification, we captured the pre- and post-polymerization states in an asymmetric unit, clearly demonstrating the in-line S_N_2 attack mechanism of the polymerization reaction. In addition, our single-selenium-atom strategy allowed the simple exploration of MeC and its catalysis in the phosphodiester bond formation. Interestingly, our studies revealed that the third ion is more critical than the other two ions in both substrate recognition and catalysis.

## 2. Results

### 2.1. Overall Structures of the Complexes

**Figure 1:**
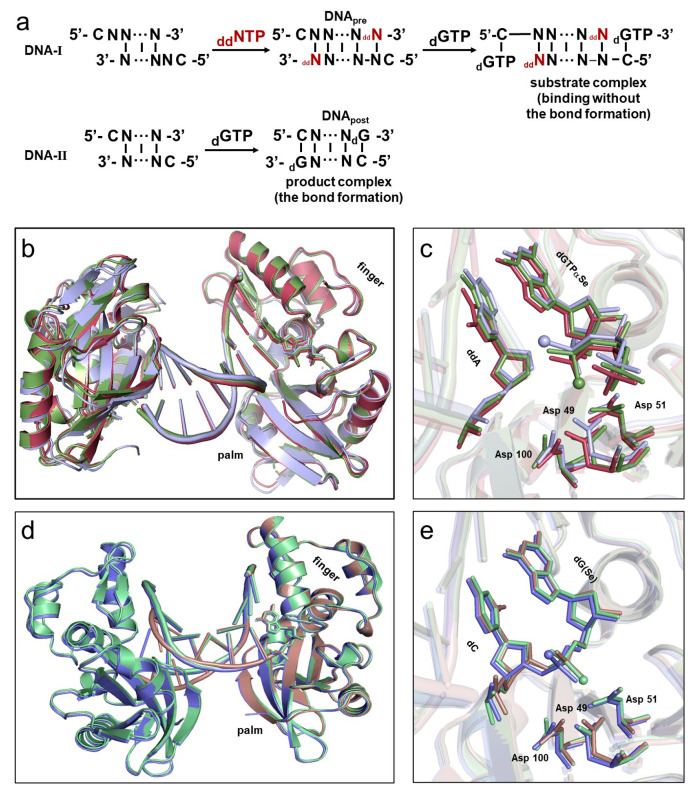
Overview of DNA polymerizations and their structures before and after the phosphate-ester-bond formation. (**a**) Chemical reaction schemes before and after the bond formation. (**b**) Superposition of three pre-polymerization state structures of the complexes: nat-dG_sub (in dark red), dGSe-I_sub (in dark green), and dGSe-II_sub (in light blue). (**c**) Superposition of the structures of the active sites and bound reactants. (**d**) Superposition of three post-polymerization state structures: nat-dG_pro (in deep purple), dGSe-I_pro (in pale green), dGSe-II_pro (in dusty blue). (**e**) Superposition of the structures of the active sites and products. The catalytic Asp residues, the incoming dGTP/dGTPαSe, and the incorporated dG/dGpSe are shown as sticks. Selenium atoms are shown as spheres.

**Figure 2:**
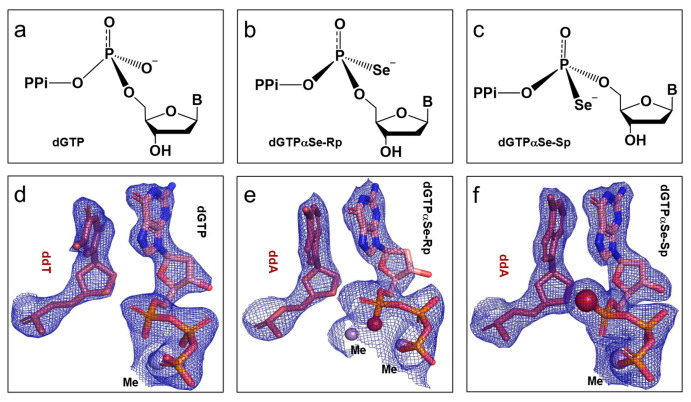
High-resolution structures of the determined triphosphates in absolute configurations. (**a**–**c**) Stereochemical formula of canonical dGTP (**a**), dGTPαSe-Rp (**b**), and dGTPαSe-Sp (**c**). (**d**–**f**) Absolute configuration identified in pre-polymerization-state complexes: dGTP (**d**), dGTPαSe-Rp (**e**), and dGTPαSe-Sp (**f**). All ddNTPs are shown as dark red sticks, and dGTP/dGTPαSe isomers are shown as sticks with Cα colored in pink. Me^2+^ and selenium atoms are shown as light-blue and firebrick spheres, respectively.

Atom-modified dNTPs (such as dNTPαSe, dNTPαS, and dNTPαB) with chiral α-P centers have been widely used to synthesize modified DNAs for therapeutic and diagnostic applications [46,47,48,49,50]. However, at the atomic level, it remains challenging to probe and elucidate the mechanisms, including catalysis, kinetic steps, and thermodynamic interactions in DNA phosphodiester bond formation and polymerization. To explore these mechanisms, we developed an atomic strategy by using modified dNTPs (such as dGTPαSe; Figure 1 and Figure 2) carrying one Se atom replacing non-bridge oxygen. Firstly, we synthesized and HPLC-purified the two dGTPαSe diastereomers [45]: dGTPαSe-I and dGTPαSe-II (Figure S1a,b). Secondly, self-complementary DNA sequences were designed with one- (DNA-II) or two-nucleotide overhang (DNA-I; Figure 1a and Figure S1c): the DNA-I allowed the incorporation of ddNTP and binding of dNTP to form the substrate complex, and the DNA-II allowed the incorporation of dNTP to form the product complex. Two DNA reactions were performed by incubating these two DNA sequences with dNTP (with or without ddNTP) separately in the polymerization system (composed of Pol X and reaction buffer). DNA_pre_, generated on the DNA-I by incorporating ddNTP without 3′-OH (Figure 1a), failed to attack the incoming dNTP (dGTP, dGTPαSe-I, or dGTPαSe-II), thereby forming the substrate complex (the substrate–primer-template–polymerase complex: nat-dG_sub, dGSe-I_sub, or dGSe-II_sub), resulting in the before-reaction state (without the bond formation) retaining the absolute configuration of dGTPαSe. In contrast, when using DNA-II (with 3′-OH) and dNTP (dGTP, dGTPαSe-I, or dGTPαSe-II), the DNA extension reaction took place to form DNA_post_, and the phosphodiester bond was formed (the after-reaction state, with the bond formation), followed by solving the absolute configuration of the Se-atom-modified P-center on DNA_post_ in the product complex (the product-template–polymerase complex: nat-dG_pro, dGSe-I_pro, or dGSe-II_pro). Finally, both dGTPαSe diastereomers were used for determining these absolute configurations, and they provided catalytic insights into the O–metal interactions and S_N_2 reaction with the configuration inversion during the bond formation.

In a series of co-crystallization trials, we meticulously examined the complex structures using the three substrates (including native dGTP and dGTPαSe diastereomers) complexed with DNA and Pol X (with or without ddNTP). Our efforts resulted in the acquisition of numerous high-quality crystals, including a total of six sets of crystals for both the substrate and product complexes, which were subsequently subjected to crystal structure determination (Table S1). These structures were refined to atomic resolution, ranging from 1.8 to 2.98 Å. As expected, all structures of the three substrate complexes captured the reaction state at the pre-polymerization level, and they belonged to the P2_1_2_1_2_1_ space group. Each asymmetric unit contained DNA duplex, dNTP substrates, and Pol X molecules symmetrically, and was structured as a three-layer sandwiched framework. Superposition showed that the three overall structures were very similar (Figure 1b,c), and their root-mean-square deviations (RMSD) were approximately 0.7–1.3 Å. On the other hand, all three product-complex structures captured the reaction state at the post-polymerization level successfully. Though one nat-dG_pro complex and two dGSe_pro complexes belonged to P2_1_2_1_2_1_ and P2_1_ space groups, respectively, their structures with the polymerase were virtually identical (Figure 1d,e), especially the two dGSe_pro structures (RMSD: 0.2 Å). We found that, in the same fashion, the phosphate of the incorporated dN interacted with three highly conserved aspartate residues (Asp49, 51, and 100) in the catalytic site.

### 2.2. The Absolute Configurations of the Two dGTPαSe Diastereomers

As the mechanism of the phosphodiester bond formation on the α-P center of dNTPs is critical for understanding polymerization reaction and designing effective drugs, we explored the atomic mechanism by replacing the dNTP α-P-non-bridge oxygen with a single Se atom (dGTPαSe; Figure 1 and Figure 2a–c). Firstly, we determined the absolute configurations of these two dGTPαSe diastereomers within the substrate complexes. In crystallization trials, DNA_pre-I_ (generated form 5′-CAGGATCCT-3′ with ddTTP, without 3′-OH) and DNA_pre-II_ (generated form 5′-CTGGATCCA-3′ with ddATP, without 3′-OH) could not be extended by attacking dGTP or dGTPαSe diastereomers in the crystallization conditions, which allowed the dNTPs to just bind and retain their absolute configuration. In the determined structures, the clear 2Fo-Fc electron-density maps indicated that these dGTP (Figure 2d) and dGTPαSe (Figure 2e,f) diastereomers were intact. As expected in their substrate structures, the Pol X active site captured dGTP or dGTPαSe coordinating with metal ions and pairing with 5′-dC on the DNA templates (Figure S2). The nat-dG_sub and dGSe-II_sub structures were refined at similar resolutions, 2.25 and 2.29 Å, respectively. However, when comparing the substrate structures containing native dGTP and dGTPαSe-II, we only found a strong Se-density difference in the electron map, which indicated the Sp configuration on the Se-modified α-P center (Figure 2f), determining the absolute configuration of dGTPαSe-II (Sp diastereomer; Figure S3c,f). Further, when we compared the substrate structures containing native dGTP and dGTPαSe-I, we only found the Se-density difference in the electron map, which indicated the Rp configuration on the Se-modified α-P center (Figure 2e), determining the absolute configuration of dGTPαSe-I (Rp diastereomer, Figure S3a). As two metal ions occupied the MeA and MeB sites, and they coordinated with the Rp atom in the dGSe-I_sub structure, the electron density on the Rp position was reduced. The distance (in the dGSe-I_sub complex) between the Rp Se atom and MeA (or MeB) was 2.3 Å (or 2.6 Å; Figure S2f), while the corresponding one (in native dGTP complex) was 2.0 Å (or 2.1 Å; Figure S2e,g), confirming the interaction between the Se atom and the metal ions. Furthermore, we found that on the phosphonoselenoate group, the P-Se bond length was 1.9 Å (single bond), while the P-O bond length was 1.6 Å [51] (double bond).

### 2.3. Configuration Inversion at the α-Phosphorus Center in the Polymerization Reaction

**Figure 3:**
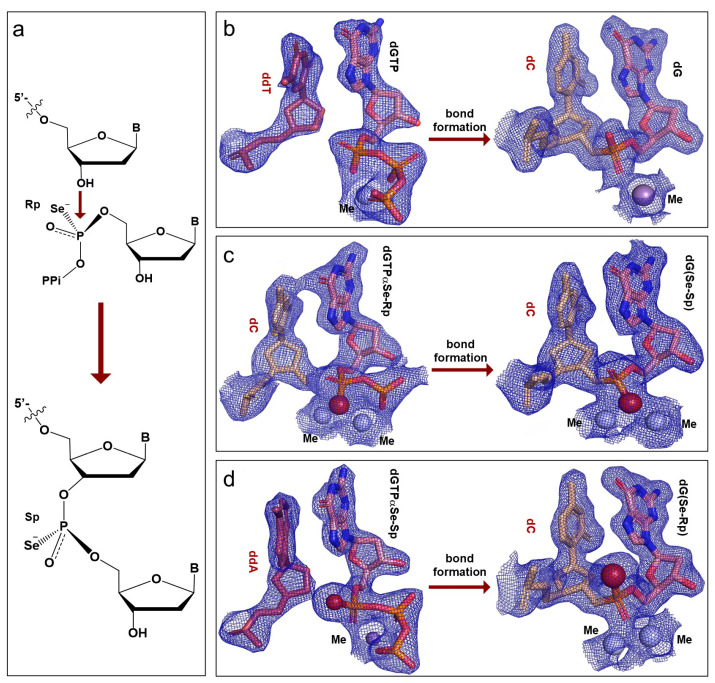
Comparison of the absolute configuration inversions using the following triphosphates: dGTP, dGTPαSe-Rp, and dGTPαSe-Sp. (**a**) The chemical reaction scheme of the inversion. (**b**–**d**) The changes in the electron-density maps before and after the bond formations (the pre- and post-polymerization states) with dGTP, dGTPαSe-Rp, and dGTPαSe-Sp, respectively. The sticks are colored the same as in Figure 2. The 2Fo-Fc electron-density maps were contoured at the 1.5 σ level. Me^2+^, and selenium atoms are shown in light-blue and firebrick spheres, respectively.

In order to elucidate the P-O bond formation mechanism in the polymerization reaction, we captured the substrate and product complexes and determined their structures at the atomic level, providing convenient and direct evidence of the inversion mechanism at the α-P center of dNTPs in the DNA synthesis (Figure 3a). In detail, we designed the crystallization trials with dGTP and dGTPαSe to form the products (DNA_post_: 5′-CGGATCC-dG-3′ and 5′-CGGATCC-dG(Se)-3′) from a self-complementary primer (5′-CGGATCC-3′) with one overhang 5′-dC-nucleotide. The 3′-OH attacked the incoming dGTP or dGTPαSe diastereomers in the crystallization conditions, thereby generating DNA_post_ and forming the native and Se-modified product complexes. The determined overall structures reached the atomic resolution, and the P-center configurations of these three product complexes (nat-dG_pro, dGSe-I_pro, and dGSe-II_pro) were solved as well (Figure 3b–d). In the determined structures, the clear 2Fo-Fc electron-density maps and polder maps indicated that the three dNTPs were individually incorporated into the three DNA_post_ products. When compared to the structure of the native product complex, the Se-modified product complex (formed from dGTPαSe-Rp) generated extra and strong electron density only for the Se atom on the P-center, revealing its Sp configuration and indicating the two-metal Se coordination (Figure 3c and Figure S3b). Similarly, the Se-modified product complex (formed from dGTPαSe-Sp) generated extra and very strong electron density only for the Se atom on the P-center, revealing its Rp configuration and indicating the two-metal O coordination (Figure 3d,f and Figure S3d). Based on these structural observations, the Se-modified P-center configurations on the two dGSe_pro structures (synthesized from dGTPαSe-Rp and -Sp) were determined as Sp and Rp, respectively, indicating the P-center inversion (Figure 3c,d).

### 2.4. The In-Line S_N_2 Attacking Observed in One Single Crystal with dGTPαSe-Rp

**Figure 4:**
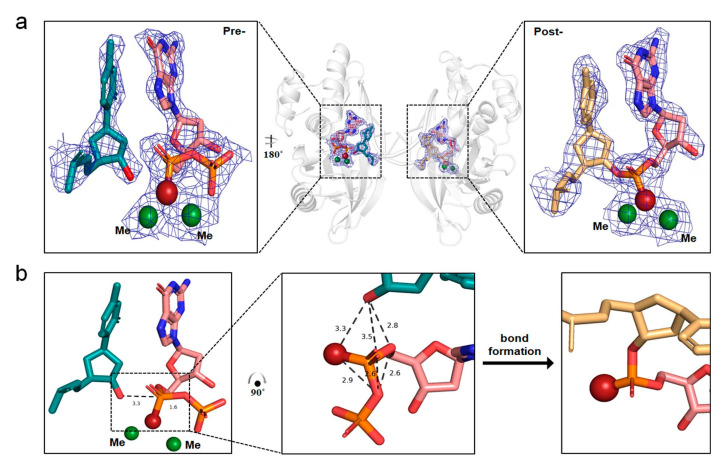
DNA polymerization followed the in-line attacking S_N_2 mechanism in a single crystal. (**a**) Close-up view of the active sites before and after the bond formation in the single-crystal dGSe-I_pro structures. (**b**) Close-up view of the trigonal bipyramid geometry of the S_N_2 in-line attacking in the DNA polymerization. Me^2+^ and selenium atoms are shown as green and red spheres, respectively. The terminal dC of primer is shown as cyan sticks with 3’-OH highlighted in red. The dGTPαSe is shown the same as in Figure 2. The dC in the extended primer is shown in bright yellow sticks. Me^2+^ and selenium atoms are shown as dark green and firebrick spheres, respectively.The electron-density maps were contoured at the 1.8 σ and 1.5 σ level before and after the bond formation, respectively.

To capture the reaction at the post-polymerization state, we designed and applied the crystallization droplets containing dGTPαSe-Rp, DNA-II, and Pol X. Surprisingly, we found that in one asymmetric unit, there was one substrate complex and three product complexes, including two DNA duplexes with four enzyme molecules. In other words, in the unit cell, the phosphoryl transfer reaction took place in three product complexes, whereas the last one was trapped in a transition-like state (Figure 4a), where the incoming dGTPαSe-Rp paired with 5′-dC of the template strand. In the substrate complex, though two Me^2+^ ions at the active center coordinated with the incoming dGTPαSe-Rp, the phosphoryl transfer reaction did not occur, indicated by the clear density gap between the primer 3′-OH and the substrate α-phosphorus. Superposition of the three post-polymerization state complexes showed that these product structures were very similar (RMSD: 0.094 Å), whereas the RMSD between the intermediate- and post-polymerization states was also similar (0.158 Å), indicating that the intermediate state was transition-like. Interestingly, the unexpected transition-like state complex revealed the in-line attacking S_N_2 mechanism in the phosphoryl-transfer polymerization, where with the attacking of the 3′-OH on the α-P center, a new bond was formed and the pyrophosphate was left [52,53]. To catalyze the reaction, two Me^2+^ ions coordinated with the 3′-OH and the incoming dGTPαSe-Rp, whereas MeA interacted with the 3′-OH and Se atom and brought them together for the bond formation (Figure 4b). The distance (3.3 Å) between the 3′-OH and the α-P atom was shorter than the relaxed distance (4.1 Å) provided in the literature [21], indicating the readiness state of the nucleophile for attacking. On the basis of the local structure (the trigonal bipyramid-like geometry) and the transition-like state, the attacking 3′-OH formed the hydrogen bond with the substrate 5′-O (indicated by their 2.8 Å distance), which facilitated the 3′-OH attacking and the phosphodiester bond formation. Both the before- and after-reaction states were captured in the same single crystal for the first time, providing new insights into the trigonal bipyramid-like geometry formed in the polymerization reaction. Clearly, our investigations of this unique crystal offered direct structural evidence of the in-line attacking S_N_2 mechanism in the bond formation [21,46,54].

### 2.5. DNA Polymerase Recognizing dNTPαSe as the Substrates in Thermodynamic and Kinetic Studies

**Figure 5:**
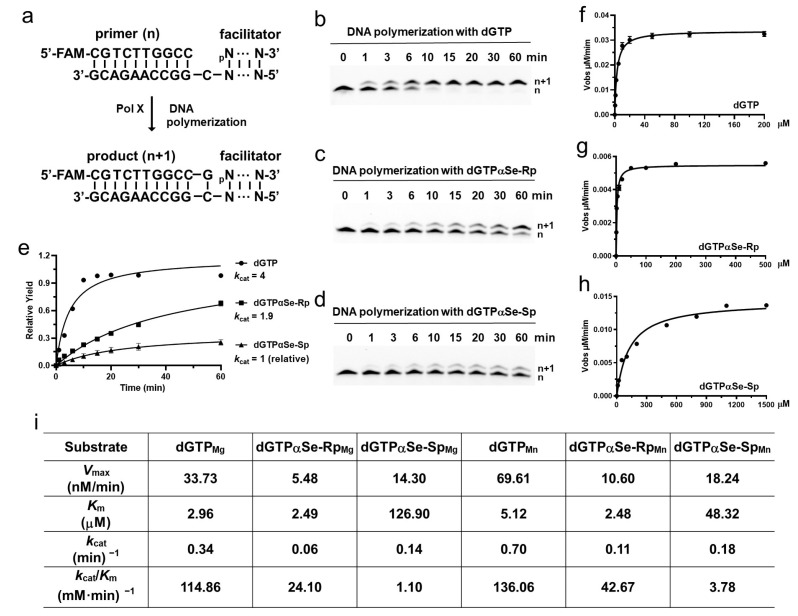
The kinetics study of Pol X using three triphosphate substrates (dGTP, dGTPαSe-Rp, and dGTPαSe-Sp). (**a**) The standing-start kinetic DNA polymerization (primer extension) (**b**) with dGTP, (**c**) with dGTPαSe-Rp, (**d**) with dGTPαSe-Sp. The apparent kinetic products of the primer extension were analyzed by denaturing PAGE, offering single-nucleotide resolution. (**e**) Kinetic analysis of (**b**–**d**) study; all *k*_cat_ values were normalized to dGTPαSe-Sp. The standard kinetic DNA polymerization with dGTP (**f**), with dGTPαSe-Rp (**g**), with dGTPαSe-Sp (**h**). (**i**) Table summary of standard kinetic constants, the constants in the presence of different metal ions are shown with the subscripts Mg or Mn.

Our structural studies indicated that both diastereomers of dNTPαSe could be recognized and incorporated into DNA by Pol X, which catalyzed the gap-filling during the DNA repair process and was consistent with our previous observation [45,55]. To investigate the dNTPαSe recognition and preference of Pol X (Figure 5), we performed the thermodynamic study with ITC (using 5′-CGGGATCC_dd_C-3′), steady-state apparent, and standard kinetic studies (using a dsDNA substrate containing a 5′-FAM-primer (5′-CGTCTTGGCC-3′), 5′-phosphate-downstream-facilitator DNA (5′-_p_CCACACCTTTACTACCTTA-3′), and template (5′-TAAGGTTAGTAAAGGTGTGGCGGCCAAGACG-3′)). Our kinetic and thermodynamic experiments with our Se-atom strategy were performed to uniquely illustrate the third-metal-ion catalytic mechanism of DNA polymerases, especially via the *k*_cat_/*K*_m_ selectivity analysis. In the presence of Mg^2+^ ions (Figure 5 and Figure S4), the *k*_cat_/*K*_m_ values of the modified dGTPαSe-Rp and dGTPαSe-Sp were 4.8 and 104.4 times smaller than that of the non-modified dGTP, respectively, illustrating that the Se-Sp atom disruption (Figure 3) of the interaction between the O-Sp atom and the third metal ion was critical for the reaction catalysis. The large difference (21.8 folds) in the enzymatic selectivity on the Se-Rp and Se-Sp diastereomers was attributed to the different impact of the Se-Rp and Se-Sp atoms on the chemical reaction, indicating that in the presence of the Sp-diastereomer, the disruption of the third metal ion by the Se-Sp atom reduced the metal-ion-enhanced catalysis significantly (Figure S6). Further, in the presence of Mg^2+^ ions, the binding affinity (*K*_m_) values of the modified dGTPαSe-Rp and dGTPαSe-Sp were 0.84 and 42.9 times larger than that of the non-modified dGTP, respectively, illustrating again that the Se-Sp atom disruption (Figure 3) of the interaction between the O-Sp atom and the third metal ion was critical for the substrate affinity and binding. The large difference (51.1 folds) in the affinities (*K*_m_) of the Se-Sp and Se-Rp diastereomers with the polymerase was attributed to the different impact of the Se-Sp and Se-Rp atoms on the binding, indicating that in the presence of the Sp diastereomer, the disruption of the third metal ion by the Se-Sp atom reduced the affinity significantly.

Similarly, in the presence of Mn^2+^ ions, our Se-atom strategy was used to uniquely illustrate the third-metal-ion catalytic mechanism of DNA polymerases, especially via the *k*_cat_/*K*_m_ selectivity analysis (Figure 5c). The *k*_cat_/*K*_m_ values of the modified dGTPαSe-Rp and dGTPαSe-Sp were 3.2 and 36.0 times smaller than that of the native dGTP, respectively, illustrating that the Se-Sp atom disruption of the interaction between the O-Sp atom and the third metal ion was critical for the reaction catalysis. The large difference (11.3 folds) in the enzymatic selectivities on the Se-Rp and Se-Sp diastereomers was attributed to the different impact of the Se-Rp and Se-Sp atoms on the chemical reaction, indicating that in the presence of the Sp-diastereomer, the disruption of the third metal ion by the Se-Sp atom reduced the metal-ion-enhanced catalysis significantly (Figure S6). Further, the *K*_m_ values of the modified dGTPαSe-Rp and dGTPαSe-Sp were 0.48 and 9.4 times that of the native dGTP, respectively, illustrating that the Se-Sp atom disruption (Figure 3) of the interaction between the O-Sp atom and the third metal ion was critical for the substrate affinity and binding. The large *K*_m_ difference (19.5 folds) in the affinities of the Se-Sp and Se-Rp diastereomers with the polymerase was attributed to the different impact of the Se-Sp and Se-Rp atoms on the binding, indicating that in the presence of the Sp-diastereomer, the disruption of the third metal ion by the Se-Sp atom reduced the affinity significantly. Therefore, in the presence of Mg^2+^ or Mn^2+^ ions, the polymerase recognizes dGTPαSe-Rp as a more favorable substrate than dGTPαSe-Sp, and our Se-atom-probing approach has uniquely demonstrated that the third metal ion is much more critical in both the binding and catalysis than the other two metal ions.

## 3. Discussion

In this study, via the single-Se-atom derivatization, we firstly created the new α-P chiral center substrates as tools in order to perform the structural study and explore the insights into the polymerase mechanism (Figure 1 and Figure S1). Further, comparing the native and Se-derivatized complex structures, we found that their active-site structures were virtually identical. Furthermore, we determined the absolute configurations (Rp and Sp) of the dGTPαSe diastereomers, via the substrate complex structures of the native and Se-modified dGTPs with Pol X and DNApre (Figure 2, Figures S2 and S3 ). In addition, the complex structures of the three products generated from the native and Se-modified dGTPs were virtually identical, and the absolute configurations of the Se-generated P-centers on the two dGSe_pro complexes from dGTPαSe-Rp and -Sp were determined as Sp and Rp (Figure 3 and Figure S3), respectively. By providing structural evidence, the P-center configuration comparison of the substrate and product complexes indicated the center inversion in the DNA-synthesis process (Figure 3). Excitingly, we captured both the before- and after-reaction states in the same single crystal for the first time, and the before-reaction state (the transition-like state; Figure 4) provided new insights into the trigonal bipyramid-like geometry formed in the polymerization reaction. Clearly, our investigations on this unique crystal offered direct structural evidence of the in-line attacking S_N_2 mechanism in the bond formation [21,46,54].

In the DNA synthesis catalyzed by DNA polymerase, three metal ions (MeA, MeB, and MeC) played key roles in the dNTP binding, activation of the primer terminal-3′-OH, and stabilizing the substrate α-P center and leaving group in the catalytic process. Since Mn^2+^ is commonly used in X-ray crystallographic studies of DNA polymerases, has a similar coordination pattern to Mg^2+^, and can be readily identified in structures [19,25,56], both divalent metal ions were used in crystal growth and are represented here as Me, while Mg^2+^ was used in the kinetic and thermodynamic studies. Our substrate complex structures indicated that MeA interacted with the 3′-OH and Rp-O on the α-P center (Figure 4 and Figure S5), whereas MeB interacted with the Rp-O on the α-P center and the non-bridging oxygens on the β center. Further, to utilize the Se atomic probe for exploring the metal-ion catalysis, we performed the thermodynamic and kinetic studies in the presence of Mg^2+^ or Mn^2+^ with the native and modified dGTPs. Our experimental results indicated that Pol X recognized both dGTPαSe diastereomers for DNA synthesis, whereas dGTPαSe-Rp was a better substrate than the Sp diastereomer (Figure 5). Clearly, our designed single-Se-atom on the dNTP substrate can atom-specifically disrupt and probe the interactions between the O atoms and metal ions, suggesting that dGTPαSe-Rp was a better substrate than dGTPαSe-Sp, indicating a stronger interaction between the Sp-O and MeC. Via kinetic and thermodynamic studies, the designed Se-atom-specific disruption provided precise insights into the binding and catalysis of the elusive third metal ion. The Se-atom in the Sp-substrate disrupted the interaction of the third metal ion, while the Rp Se-atom did not, which caused the Sp *k*_cat_/*K*_m_ (catalytical efficiency) to be smaller than the Rp one. Our studies revealed that the MeC metal ion is much more critical than the other two ions in both substrate recognition and catalysis (Figure 5 and Figure S6). Therefore, our thermodynamic, kinetic, and structural studies demonstrate that our new Se-atomic strategy is much easier for investigating the mechanisms than the time-resolved strategy [20,23,24,57], especially for targeting DNA polymerases and developing drugs for treating cancers and other diseases [1,58].

## 4. Materials and Methods

### 4.1. Protein Expression and Purification

Pol X (Uniport code: P42494) was cloned to pET28a-Sumo, expressed in *Escherichia coli* BL21(DE3), and purified according to the described method [7]. Briefly, the recombinant strains were revived in Lysogeny broth (LB) medium with kanamycin (final concentration: 50 μg/mL) at 37 °C overnight, followed by its inoculation (ratio 1:100) in new LB medium (with 50 mg/mL kanamycin) and incubation at 37 °C with continuous shaking. When the culture OD600 reached approximately 0.7, the Pol X expression was induced with isopropyl β-D-1-thiogalacto-pyranoside (IPTG, 0.2 mM), followed by cultivation at 16 °C for 24 h. Then, the cells were harvested by centrifugation (8000× *g*) at 4 °C for 25 min. 

The cell pellets were resuspended in buffer A (20 mM Tris pH 8.0, 500 mM NaCl, 25 mM imidazole) and lysed under high pressure via a cell crusher (Scientz-150). The ensuing homogenate was clarified by centrifugation (12,000× *g*) at 4 °C for 40 min and the supernatant was loaded onto a HisTrapTM HP column (GE Healthcare, Chicago, IL, USA) equilibrated with buffer A. Pol X was eluted from the column using buffer B (20 mM Tris, pH 8.0; 500 mM NaCl; 500 mM imidazole) with a gradient (0–100%). The fractions containing the desired amounts were pooled, and buffer B was exchanged with buffer A, at 4 °C by dialysis with buffer A. Then, the His-Sumo tag of the fused Pol X was removed by a ULP1 protease cleavage, followed by his-tag affinity purification. After the affinity-purified sample was diluted with the binding buffer (20 mM Tris, pH 8.0; 100 mM NaCl), it was applied to a HisTrap SP HP column (for strong cation-exchange chromatography, GE Healthcare), followed by elution using the buffer (20 mM Tris, pH 8.0; 1 M NaCl) with a gradient of (0–100%). The collected portions were concentrated with an ultrafiltration tube (Millipore, Burlington, MA, USA) and loaded onto a Hi 16/60 Superdex G75 column (GE Healthcare) equilibrated with gel-filtration buffer (20 mM Tris-HCl, pH 8.0; 100 mM NaCl; 1 mM DTT). Finally, purified Pol X was quantified and stored in a solution of glycerol (20%), NaCl (500 mM), and DTT (2 mM) at −80 °C.

### 4.2. Crystallization and X-ray-Diffraction Data Collection

All crystallization trials were prepared by mixing Pol X, DNA, MnCl_2_ or MgSO_4_, and dGTP or dGTPαSe (with or without ddNTP) on ice. Gryphon crystallization robot system (Art Robbins Instrument, San Francisco, CA, USA) and commercial crystallization kits (Hampton Research, Aliso Viejo, CA, USA) were used to screen for crystallization conditions at 16 °C. The sitting-drop vapor diffusion method with 3-drop Intelli-Plates was utilized during the initial screening. After the identification of initial crystallization conditions, final conditions were optimized with the hanging-drop vapor diffusion method at 16 °C, and the compositions of the final crystallization conditions are listed in Table S2.

All the complex crystals were snap-frozen in liquid nitrogen by cryoprotecting in their mother liquor supplemented with 20–30% glycerol. The X-ray diffraction data were collected on beamline BL17U, BL18U, and BL19U at the Shanghai Synchrotron Radiation Facility (SSRF) at cryogenic temperatures and maintained with a cryogenic system. Data processing was carried out using the iMosflm program (version 7.0.044) embedded in the CCP4i suite (version 7.0.044) or the HKL2000 (version 706) or HKL3000 (version 712) programs.

### 4.3. Structure Determination and Refinement

The diffraction data were indexed, scaled, and reduced using XDS (version Jan 26 2018) [59].The complex structures were solved by the molecular replacement method using the DNA:Pol X complex structure (PDB code: 5HRB) as a search model using COOT (version 0.8.9.2) [60]. The data were refined using the refine program of Phenix (version 1.13) [61] and the Refmac5 program of CCP4i [62], which revealed the detailed orientations of the missing protein residues and DNA molecules. Me^2+^ ions, water, dGTPs, and other molecules were all built manually using COOT. All structure images were created with PyMOL (version 2.5.2).

### 4.4. dNTP Binding Assay In Vitro

Isothermal titration calorimetry (ITC) assay was performed to measure the binding affinity of dGTP, dGTPαSe-Rp, and dGTPαSe-Sp to Pol X. All buffers used in the ITC experiments had the same formulation (NaCl (100 mM) and sodium phosphate buffer (20 mM, pH 8.0), MgSO_4_ (10 mM)). The ITC experiments were carried out on the MicroCal ITC200 System (Malvern Instruments, Malvern, UK) with the parameter settings as follows: syringe concentration (0.2 mM), cell concentration (0.02 mM), stirring speed (1000 rpm), injection volume (2 μL), injection duration (2 s), total injections (20 times), cell temperature (37 °C), reference power (10 μkal/s), initial delay (60 s), and injection spacing (90 s). The mixture of Pol X (0.02 mM) and DNA_dd_C (0.04 mM) was first added to the sample cell, and a syringe was filled with dGTPs (0.5 mM). The data were further analyzed using the Origin 7.0 software, with background subtraction using the last few titration data.

### 4.5. The Apparent and Standard Kinetic Study of Polymerase

Apparent kinetic and standard kinetic assays on two dGTPαSe diastereomers were performed to explore the reaction rate and selectivity of Pol X. The DNA assay system (1-nt-gap DNA_P_) was prepared by heating and incubating 5′-Fam-Tagged primer, 5′-phosphate-Tagged downstream and standing-start template to 95 °C and cooling to room temperature slowly. For the apparent kinetic assay, the reaction mixture (50 μL) contained Tris-HCl (50 mM, pH 7.8), NaCl (50 mM), DTT (1 mM), bovine serum albumin (BSA, 0.01% *w*/*v*), MgCl_2_ (10 mM), Pol X (0.2 μM), 1-nt gap DNA_P_ (5 μM), and dNTPs (150 μM). The liquid (5 μL) was collected from the solution incubated at 37° for 0, 1, 3, 6, 10, 15, 20, and 30 s time intervals, followed by quenching with an equal volume of 2× loading buffer (10 M urea; 30% glycerol; 0.01% Xylene Cyanole FF; 20 mM EDTA). For the standard kinetic assay, the reaction mixture (10 μL) contained Tris-HCl (final concentration: 50 mM pH 7.8), NaCl (50 mM), DTT (1 mM), bovine serum albumin (BSA, 0.1 mg/mL), MgCl_2_ (10 mM), PolX (0.1 μM), 1-nt gap DNA_P_ (5 μM), and the concentration of dGTP (0.5, 1, 2, 5, 10, 20, 50, 100, 200 μM), dGTPαSe-I (1, 2, 5, 10, 20, 50, 100, 200, 500 μM), or dGTPαSe-II (10, 20, 50, 100, 200, 500, 800, 1100, 1500 μM). The mixtures were incubated at 37 °C for 5 min and quenched with an equal volume of 2× loading buffer (10 M urea, 30% glycerol, 0.01% Xylene Cyanole FF, 20 mM EDTA). All apparent and standard kinetic samples were further terminated by being heated to 95 °C for 5 min and immediately placed on ice, analyzed by 25% polyacrylamide gels containing urea (6 M) at 55 °C, and quantified with ImageJ software (version 1.52a). Quantification of *V*_max_, *K*_m_, and *k*_cat_ value, and graphic representation and fitting were executed by Graph Prism with a Michaelis–Menten model.

## 5. Conclusions

We determined the structures of the substrate and product complexes using native or Se-modified dGTPs, and our structural studies solved the absolute configurations of the dGTPαSe diastereomers for the first time, providing a new α-P-center-chiral substrate probe for exploring the mechanism of phosphodiester bond formation in DNA synthesis. In addition, our thermodynamic and kinetic studies indicated that DNA polymerase can effectively recognize both diastereomers, whereas dGTPαSe-Rp is a better substrate. Further, we provided simple and direct structural evidence of the α-P-center inversions during DNA polymerization, assisting the stereochemistry study, exploring the mechanism of DNA synthesis, and establishing a direct strategy via single-Se-atom substitution. Furthermore, with our new strategy, capturing the before- and after-reaction states in the same single crystal, we illustrated the trigonal bipyramid-like geometric in the phosphoryl transfer reaction, offering convenient structural evidence of the in-line attacking S_N_2 mechanism of DNA polymerization. Moreover, by integrating thermodynamic and kinetic studies with different metal ions, we demonstrated that MeC played a much more critical role than the other two metal ions, in both dNTP binding and bond-forming catalysis, thereby providing new insights for designing polymerase inhibitors. In general, our single-Se-atom strategy opens new research avenues for DNA synthesis, DNA polymerization sequencing, and better drug design in targeting the polymerases, and it also has great potential in exploring other mechanisms and enzymes, such as ligases and kinases working with dNTPs and NTPs.

## Data Availability

All the complex structures reported in this article have been deposited in the Protein Data Bank under accession codes 8ILD, 8ILG, 8ILE, 8ILH, 8ILF, and 8ILI for nat-dG_sub, nat-dG_pro, dGSe-I_sub, dGSe-I_pro, dGSe-II_sub, and dGSe-II_pro, respectively. All other data are available from the authors upon reasonable request.

## Supplementary Materials

The supporting information can be downloaded at https://www.mdpi.com/article/10.3390/ijms242115758/s1.
