# Supplementary material for: Structural Insight into Polymerase Mechanism via a Chiral Center Generated with a Single Selenium Atom
Source: Int J Mol Sci. 2023 Oct 30;24(21):15758. doi: 10.3390/ijms242115758 (PMC10647396; doi:10.3390/ijms242115758)
Supplement: Supplementary file 1 [file ijms-24-15758-s001.zip › ijms-2650287-supplementary.pdf]

Supporting Information

Table S1 Data collection and refinement statistics of the complex structures

Structure	nat-dG_sub	nat-dG_pro	dGSe-I_sub	dGSe-I_pro	dGSe-II_sub	dGSe-II_pro
(PDB ID)	(8ILD)	(8ILG)	(8ILE)	(8ILH)	(8ILF)	(8ILI)
**Data collection**						
Space group	$P2_12_12_1$	$P2_12_12_1$	$P2_12_12_1$	$P2_1$	$P2_12_12_1$	$P2_1$
Cell dimensions						
a, *b*, *c* (Å)	58.7, 75.4, 108.5	69.0, 81.4, 92.6	59.5, 77.4, 111.3	60.5, 87.3, 87.1	59.7, 78.2, 101.3	60.4, 87.3, 87.5
α, β, γ (°)	90.0, 90.0, 90.0	90.0, 90.0, 90.0	90.0, 90.0, 90.0	90.0, 91.2, 90.0	90.0, 90.0, 90.0	90.0, 91.2, 90.0
Wavelength (Å)	0.979	0.979	0.979	0.979	0.979	0.979
Resolution (Å)	30.0-2.0 (2.07-2.0)	30.0-1.8 (1.86-1.8)	30.0-3.0 (3.11-3.0)	30.0-2.1 (2.18-2.10)	30.0-2.3 (2.38-2.3)	30.0-1.9 (1.97-1.9)
Completeness (%)	93.5 (92.2)	97.6 (94.1)	99.9 (100.0)	96.9 (87.2)	99.9 (99.9	97.1 (81.4)
Redundancy	5.1 (4.0)	4.4 (2.9)	12.7 (12.8)	5.0 (3.4)	9.3 (8.6)	5.0 (3.4)
$I / \sigma I$	26.6 (1.8)	17.0 (1.9)	21.0 (2.1)	19.8 (3.6)	15.0 (3.2)	25.2 (3.5)
Rmerge (%)	6.3 (67.9)	12.0 (39.8)	10.9 (61.8)	7.0 (23.7)	14.9 (70.0)	5.1 (26.2)
**Refinement**						
Resolution (Å)	28.52-2.25	28.9-1.8	30.0-3.0	30.0-2.1	30.0-2.30	30.0-1.9
R_{work} / R_{free}	21.2/26.3	22.2/25.5	22.0/28.3	18.1/22.7	20.7/24.5	21.5/24.3
No. atoms						
Protein	2763	2825	2650	5704	2840	5776
DNA/Ligand	420	644	358	622	358	644
Mn^{2+} or Mg^{2+}	2	2	4	8	2	8
B-factors						
Protein	52.7	24.7	33.4	25.6	31.3	23.2
DNA/Ligand	40.2	31.1	22.0	20.8	22.0	15.8
Mn^{2+} or Mg^{2+}	54.3	21.8	88.6	16.3	20.7	12.9
R.m.s. deviations						
Bond lengths (Å)	0.006	0.008	0.009	0.008	0.008	0.007
Bond angles (°)	1.164	0.960	1.416	1.403	1.385	1.226
Ramachandran plot						
Most favored (%)	94.5	98.6	93.2	97.3	95.7	96.1
Allowed (%)	5.5	1.4	6.8	2.7	4.3	3.9

Table S2. Sample composition and crystallization condition

Complex structure	Sample composition	Crystallization condition
nat-dG_sub	Pol X (0.2 mM)<br>ddTTP (0.75 mM)<br>dGTP (0.9 mM)<br>DNA_{pre-I} (0.5 mM)<br>$MnCl_2$ (5 mM)<br>NaCl (125 mM/drop)	Ammonium acetate (0.2 M)<br>BIS-Tris pH=5.5 (0.1 M)<br>20% PEG8000
nat-dG_pro	Pol X (0.2 mM)<br>dGTP (0.89 mM)<br>DNA_{post} (0.5 mM)<br>$MnCl_2$ (5 mM)<br>NaCl (125 mM/drop)	Potassium Formate (0.2 M)<br>16% PEG3350
dGSe-I_sub	Pol X (0.2 mM)<br>ddATP (0.75 mM)<br>dGTPαSe-Rp (0.4 mM)<br>DNA_{pre-II} (0.5 mM)<br>$MnCl_2$ (5 mM)<br>NaCl (125 mM/drop)	0.2 M L-proline<br>HEPES pH 8.6 (0.1 M)<br>10% PEG 3350
dGSe-I_pro	Pol X (0.05 mM)<br>dGTPαSe-Rp (0.13 mM)<br>DNA_{post} (0.23 mM)<br>$MgCl_2$ (5 mM)<br>NaCl (125 mM/drop)	ammonium acetate (0.2 M) HEPES pH 7.2 (0.1 M)<br>25% PEG3350
dGSe-I_sub	Pol X (0.2 mM)<br>ddATP (0.75 mM)<br>dGTPαSe-Sp (1.4 mM)<br>DNA_{pre-II} (0.5 mM)<br>$MnCl_2$ (5 mM)<br>NaCl (125 mM/drop)	ammonium sulfate (0.2 M)<br>Bis-Tris pH 5.5 (0.1 M)<br>20% PEG8000
dGSe-II_pro	Pol X (0.05 mM)<br>dGTPαSe-Sp (1.15 mM)<br>DNA_{post} (0.13 mM)<br>$MgCl_2$ (5 mM)<br>NaCl (125 mM/drop)	ammonium sulfate (0.2 M)<br>Bis-Tris pH 6.5 (0.1 M)<br>20% PEG3350

a

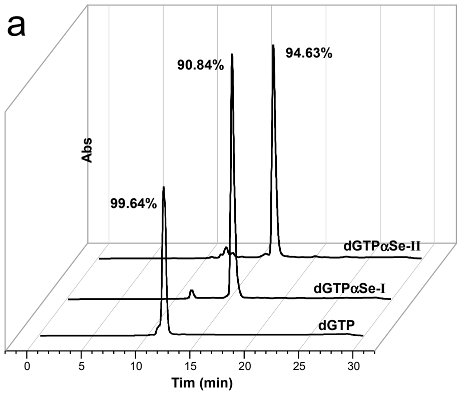

b

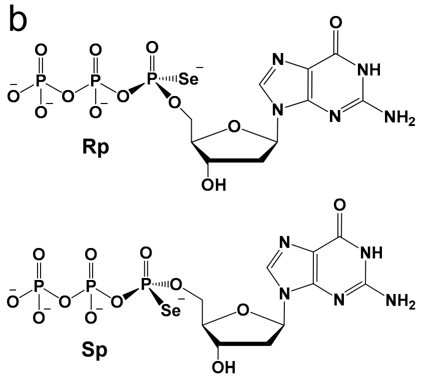

c

complex structure	DNA sequence in structure
nat-dG_sub	5'- CAGGATCCTG -3'<br>3'- GTCCTAGGAC -5'
dGSe-I_sub	5'-CTGGATCCAG-3'<br>3'-GACCTAGGTC-5'
dGSe-II_sub	5'-CTGGATCCAG-3'<br>3'-GACCTAGGTC-5'
nat-dG_pro	5'-CGGATCCG-3'<br>3'-GCCTAGGC-5'
dGSe-I_pro	5'-CGGATCCG-3'<br>3'-GCCTAGGC-5'
dGSe-II_pro	5'-CGGATCCG-3'<br>3'-GCCTAGGC-5'

Figure S1. HPLC purify of dGTPαSe diastereomers and DNA details in structures. (a) Two synthesized dGTPαSe diastereomers were separated by HPLC were named as dGTPαSe-I (with shorter retention time: 15.18 min) and dGTPαSe-II (with the longer retention time: 16.23 min), respectively, and both of them eluted later than dGTP (retention time: 11.44 min). The purity of the three compounds were marked to their UV absorption curve. (b) Stereochemical formulas of dGTPαSe diastereomers. (c) The detailed DNAs structures captured in the complex crystals. The bases lacking 3'-OH group were marked in red. dGTPαSe-Rp and dGTPαSe-Sp diastereomers captured in substrate complex structures were colored in lightblue and lightgreen bolded front, respectively. dG(Se-Sp) and dG(Se-Rp) diastereomers in the product complex structures were colored darkblue and darkgreen bolded front, respectively. The dC with 3'-OH group was colored in cyan.

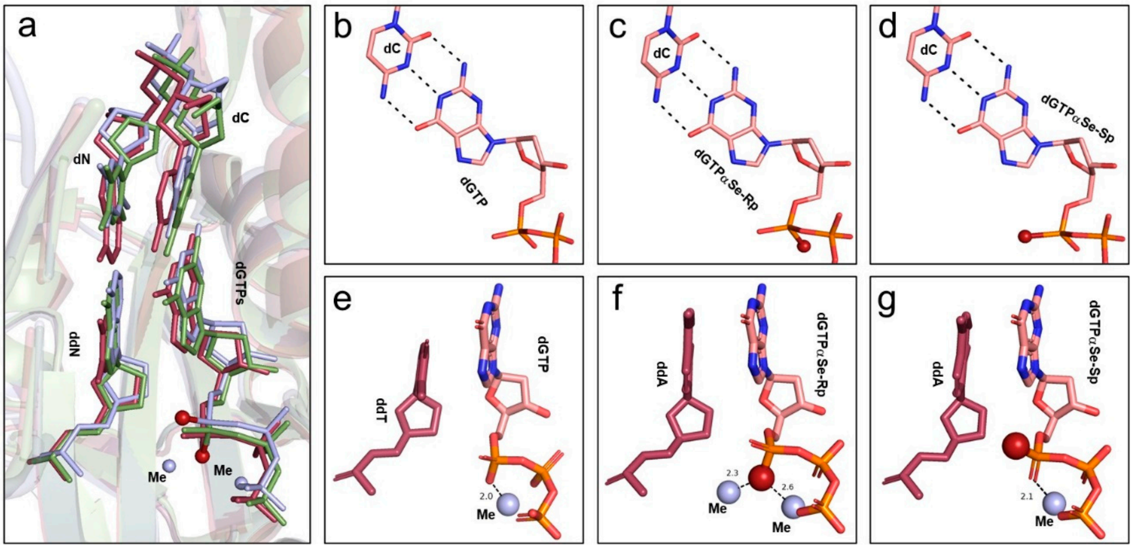

Figure S2. The binding dNTP coordinated with template and Me^{2+} in the substrate complex structures. (a) Superposition of the substrate complexes structures at the active sites. (b-d) reactants dGTPs pairing with 5'-dC on the DNA templates of dGTP (b), GTPαSe-Rp (c) and dGTPαSe-Sp (d). e-g, the Rp O/Se-atom was coordinated with Me ions of dGTP (e), GTPαSe-Rp (f) and dGTPαSe-Sp (g). All ddNTPs were shown as raspberry sticks, dGTPs and comparing 5'-dC were shown as sticks with Cα colored in pink. Se-atoms and Me^{2+} were shown as firebrick and lightblue spheres, respectively.

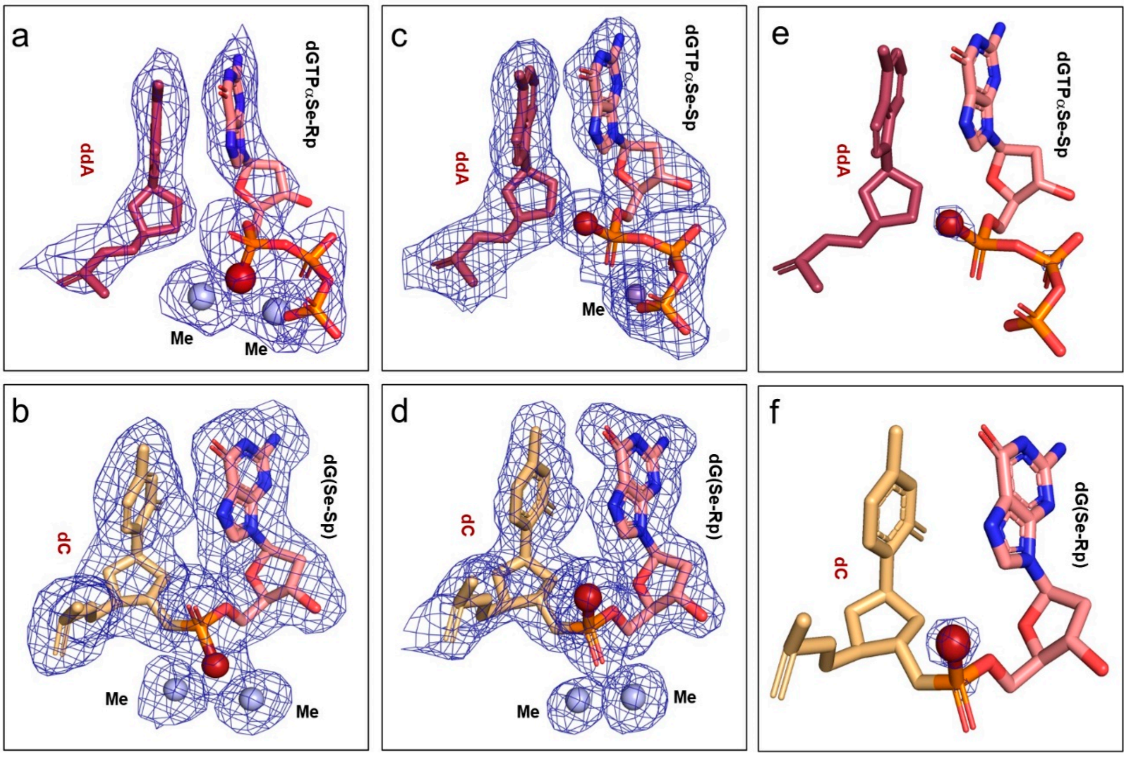

Figure S3. The polder maps of substrate dGTPαSe diastereomers and the formed product, and the map of dGTPαSe-Sp in pre- and post-polymerization states. (a-b) The polder map of dGTPαSe-Rp diastereomer in pre- (a) and post-polymerase state (b). (c-d) The polder map of dGTPαSe-Sp diastereomer in pre- (c) and post-polymerase state (d). The density map is contoured at 3 σ. Se-atoms and Me^{2+} were shown as firebrick and lightblue spheres, respectively. (e-f) maps of the dGTPαSe-Sp in the pre-polymerase state (e) and post-polymerase state (f), and the map is contoured at 15 σ.

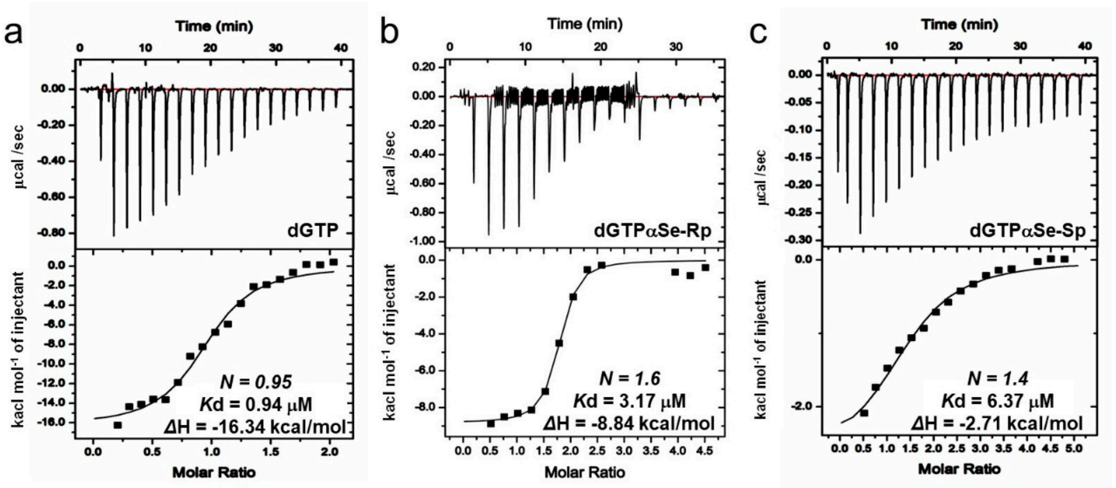

Figure S4. Selenium atom substitution affects the binding of dGTPαSe analogs to Pol X. (a-c) Isothermal titration calorimetry (ITC) analysis results showing the binding of dGTP (a), dGTPαSe-Rp (b) and dGTPαSe-Sp (c), respectively.

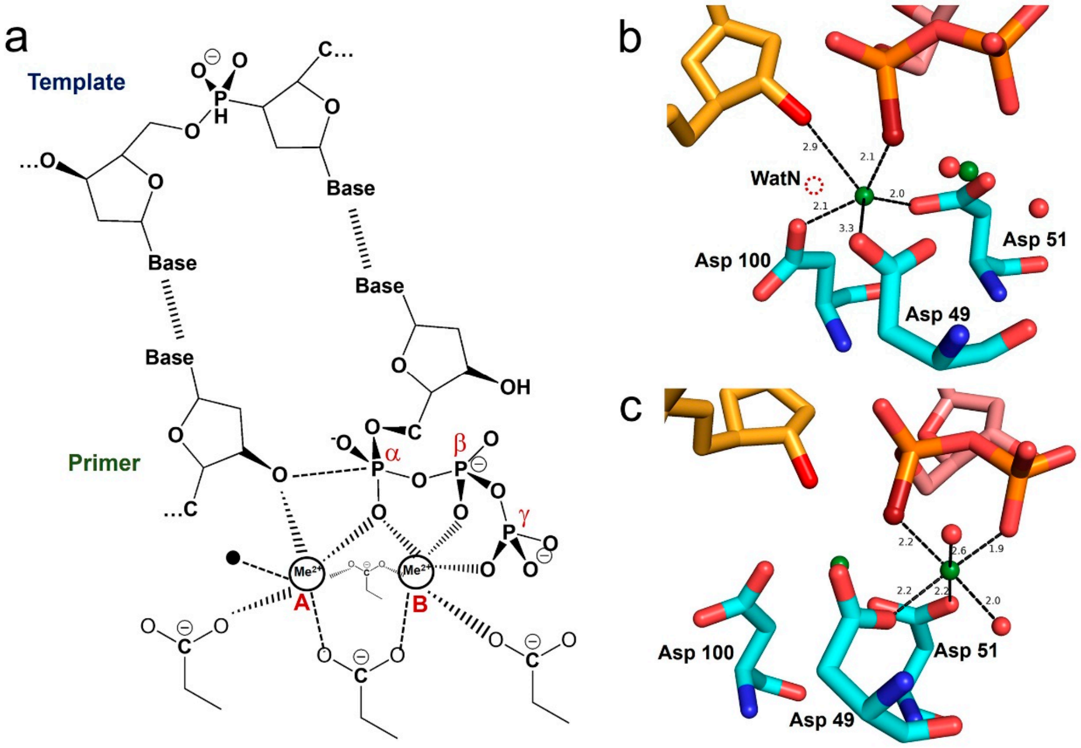

Figure S5. Coordination of two metal ions in the polymerization reaction. (a) Schematic diagram of the two-metal-ion mechanism, with MeA and MeB shown as solid circles. (b-c) The two-metal-ion coordination was observed in Pol X, with MeA (b) and MeB (c). The three Asp residues and the two nucleotides are shown as sticks. Me^{2+}, selenium atom and water molecules are shown as the spheres, colored in forest, firebrick and red, respectively.

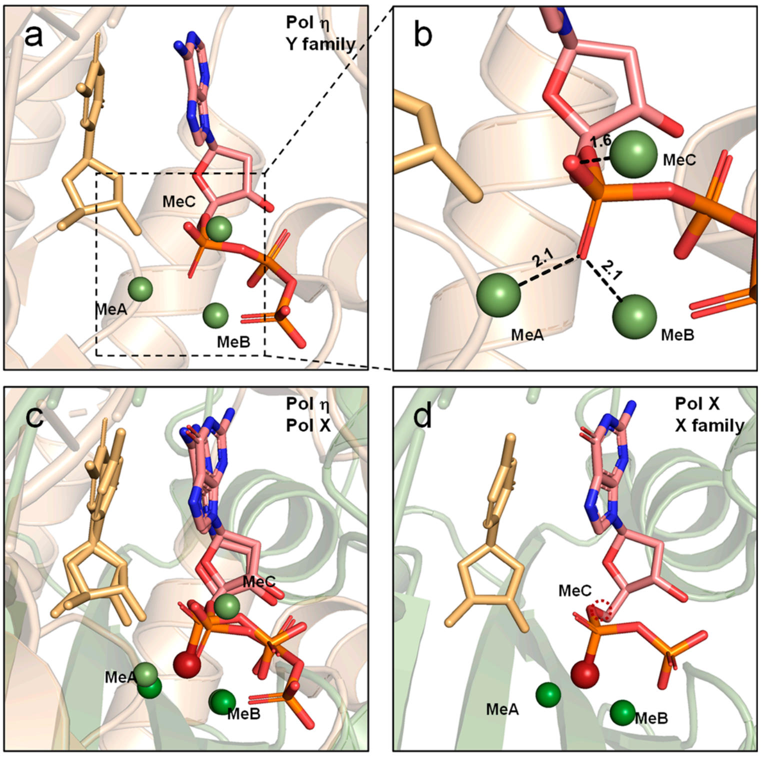

Figure S6. The three metal ions coordination in DNA polymerases. (a) the three-metal-ion coordination in the Y-family DNA polymerase (Pol η). (b) the zoom-in structure of the incoming-dNTP α-P center in (a). (c) superposition of the catalytic sites of Pol η and Pol X. (d) the hypothetic three-metal-ion coordination in Pol X. 3'-dNs of primers were shown as lightorange sticks, and the incoming dNTPs were shown as sticks with the Cα colored in deep salmon. The metal ions in Pol η and Pol X were shown as the darkgreen and forest spheres, respectively.
